# Genetic analysis of allogenic donor cells after successful allo-limbal epithelial transplantation in simple and cultivated limbal epithelial transplantation procedures

**DOI:** 10.1038/s41598-023-31261-z

**Published:** 2023-03-15

**Authors:** Suksri Chotikavanich, Nitikorn Poriswanish, Angkoon Luangaram, Parwana Numnoi, Ranida Thamphithak, Warinyupa Pinitpuwadol, Mongkol Uiprasertkul, Chareenun Chirapapaisan, Rosanun Sikarinkul, Pinnita Prabhasawat

**Affiliations:** 1 Department of Ophthalmology, Faculty of Medicine Siriraj Hospital, Mahidol University, Bangkok, Thailand; 2 Department of Forensic Medicine, Faculty of Medicine Siriraj Hospital, Mahidol University, Bangkok, Thailand; 3 Department of Pathology, Faculty of Medicine Siriraj Hospital, Mahidol University, Bangkok, Thailand

**Keywords:** Eye diseases, Stem-cell niche, Mechanisms of disease, Stem-cell research

## Abstract

This non-comparative cohort study investigated long-term donor cell survival after allogenic simple/cultivated limbal epithelial transplantations (allo-SLET/allo-CLET, respectively) by genetic analysis. Transplanted corneal epithelial cells, which underwent impression cytology and/or corneal-button biopsy, were examined for personal identities of autosomal short-tandem repeats; the percentages of donor cells were calculated based on matching recipient or donor buccal-DNA references. Twelve patients were included; 4 underwent allo-CLET, 8 underwent allo-SLET. Eight patients (67%) had total limbal stem cell deficiency (LSCD). Genetic analysis was performed postoperatively (mean, 55.3 months). Donor cells were detected in 4 of 12 patients (25%), all of whom underwent allo-SLET; 1 patient had a donor genotype and 3 patients had a mixed donor/recipient genotype. The longest time of donor cell detection was 30 months. Seven patients (58%) used systemic immunosuppressives at the time of genetic analysis (mean use, 22.5 months). Allogenic donor cells survived in both procedures for the long term postoperatively, which encourages the long-term use of systemic immunosuppressives. Donor cells may not be the only factor in graft survival, in that most successful cases had a recipient profile. Their presence for a specific time may promote niches for the patients’ own cells to repopulate, especially for partial LSCD.

## Introduction

Limbal stem cell deficiency (LSCD) is a severe ocular surface condition in which the limbal stem cells (LSCs) cannot maintain a healthy corneal epithelium, leading to conjunctival invasion onto the corneal surface. Limbal stem cell transplantation is the mainstay treatment to restore the corneal epithelium. Conventional surgeries^1^, including keratolimbal allograft (KLAL) and conjunctivolimbal allograft (CLAL), require a large amount of donor limbal tissue.

A significant advance in the field was introduced in 1997^2^, when cultivated limbal epithelial transplantation (CLET), which requires the harvesting of only a small amount of donor limbal tissue, was described. However, this technique requires expensive laboratory cell culture facilities. Simple limbal epithelial transplantation (SLET), introduced in 2012^3^, requires only diced limbal tissue expanded in vivo on the corneal surface, thus eliminating the cost of laboratory facilities. During this novel era of limbal stem cell transplantation, the two techniques were among the most commonly performed worldwide^4,5^.

Bilateral LSCD is especially challenging because allogeneic limbal epithelial transplantation (allo-LET) is required, and postoperative immunosuppression is necessary to prevent allograft rejection. Because the appropriate duration of using systemic immunosuppressive medications is not definitive^6^, this should be balanced between their various side effects^7–10^ and graft survival. In addition, the optimal site of origin of the corneal epithelial cells covering the entire corneal surface in the successful cases also is not definitive. It is interesting to explore if the cells originating from the donor gradually disappear and are ultimately replaced by the recipient cells.

To our knowledge, only two studies have reported allogenic donor survival after CLET^11,12^, and no previous studies of SLET have been published. The aim of the current study was to investigate the persistence of donor epithelial cells over the long term in patients who underwent allo-LET transplantation in which we used a new method of specimen collection and genetic analysis.

## Methods

This non-comparative cohort study followed the tenets of the Declaration of Helsinki and the institutional review board of Faculty of Medicine Siriraj Hospital, Mahidol University (approval number, Si194/2018) approved the study. All patients provided informed consent before enrollment. The study was registered in Thai Clinical Trials Registry (identification number, CTR20180510003). At the time of registration, the protocol with a sample size of 20 and a minimum follow-up of 3 months postoperatively was initially planned. However, we later extended the follow-up time to gain information on DNA analysis in the long term. Eventually, 12 patients were recruited.

The inclusion criteria included patients with bilateral LSCD who had undergone allo-CLET or allo-SLET with a follow-up of at least 1 year, age of at least 18 years, and a successful outcome defined by slit-lamp examination and laboratory investigation. Clinical success was determined by the absence of epithelial defects, absence of conjunctivalization in the central 5-mm cornea, and no/mild ocular surface inflammation. Moreover, laboratory investigations for phenotypic identification to prove the existence of corneal epithelial cells by in vivo confocal microscopy (IVCM) and/or impression cytology with immunofluorescence staining (ICIF) were included. The full protocols of both investigations were reported previously^13–15^. Briefly, the criteria included the presence of total or predominant corneal epithelial cells in the central cornea by IVCM and/or the presence of total or predominant specific markers of corneal epithelial cell (CK12) by ICIF.

The specimen collection techniques for genotyping analyses were from impression cytology (IC) and/or the superficial layer of the corneal button of penetrating keratoplasty (PK). The IC was the mainstay of the sampling in most patients. However, some patients with deep corneal stromal opacity required PK after allo-LET, and superficial dissection for the corneal epithelial layer of the corneal buttons at the time of PK was an alternative sampling technique.

The genotyping reference profiles of recipients and donors were obtained using buccal swabs. With allo-SLET, all underwent living-related allo-SLET, so all donor-reference samples were received from living relatives. However, in allo-CLET, all donors were from cadavers and the donor-reference sample collection was impossible. To avoid false donor-positive results, a buccal swab also was obtained from all specimen collectors and laboratory technicians involved in all processes. The genotypic result was interpreted as donor, recipient, or mixed (donor and recipient) genotype, depending on the matching of the results between the patients and the references after DNA genotypic analysis.

### IC technique

Corneal epithelial cell samples were collected by IC using Biopore membrane (PICM 01250, Millipore Corp., Bedford, MA, USA). After application of tetracaine hydrochloride 0.5% eye drops, the membrane was pressed lightly on the entire corneal surface for about 5 s. The procedure was performed using a sterile technique with a negative control, during which another membrane was left in the air and later underwent the same DNA genotypic analysis process.

### DNA genotypic analysis

*DNA extraction* The IC specimen and the buccal swab from the recipient, living-related donor for reference profiles, and controls were extracted using a QIAamp DNA Micro Kit (QIAGEN, Germany) and subsequently quantified by spectrophotometry.

*PCR amplification* 16 loci, each containing a polymorphic short tandem repeat, of the DNA samples were amplified following the manufacturer’s instructions for the AmpFlSTR Identifiler Plus PCR Amplification Kit (Applied Biosystems, Waltham, MA, USA). The 16 loci were D451179, D21511, D75420, CSF1PO, D381368, TH01, IM38317, IM88038, H781338, D195433, uWA, TPOX, 010561, AMH, D58818, and HGA.

*DNA genotyping* The PCR amplification product was separated by capillary electrophoresis using ABI 3500 Genetic Analyzers (Applied Biosystems) according to the instruction manual. GeneMapper ID-X Software (Applied Biosystems) was used for data analysis.

## Results

Twelve eyes of 12 patients (7 women, 5 men; mean age, 47.0 ± 16.4 years; range 27–71) who underwent allo-LET with successful outcomes were included. Eight (67%) and four (33%) eyes had been treated with living-relative-allo-SLET and allo-CLET, respectively. The demographic data and clinical characteristics of the patients are shown in Table 1. Eight patients (67%) had total LSCD. The causes of LSCD included chemical burns (4 eyes, 33%), Stevens-Johnson syndrome/toxic epidermal necrolysis) (3 eyes, 25%), aniridia (2, 17% eyes), and idiopathic causes (3 eyes, 25%). All patients had clinically successful outcomes with a relatively clear central corneal 5-mm zone and corneal epithelial cells detected at the central cornea by IVCM and/or specific markers of corneal epithelial cell (CK12) by ICIF. The visual acuity improved consistently in most patients after allo-LET and subsequent PK or cataract extraction surgeries. After allo-LET, all patients were treated with topical 1% methylprednisolone hourly, which was tapered to 4 times daily and switched to a mild steroid once daily perpetually. Systemic prednisolone 1 mg/kg/day was prescribed and tapered over 3 months. The immunosuppressive medications were mycophenolate mofetil with starting doses of 2 g/day or cyclosporine with a starting dose of 3–5 mg/kg/day before tapering. The postoperative time at which genotypic analysis was performed, when IC and/or corneal buttons were collected, was at 55.3 ± 46.6 months (range, 12–138). However, the durations of medication administration were shorter at 22.5 ± 17.3 months (range, 5–56). Although 7 patients (58%) still used medications at the time of the genetic analysis, one of whom had a history of graft rejection, the medications could be discontinued with a satisfactory ocular surface achieved in 5 patients (42%) before the genetic analysis (Table 1).

**Table 1:** Demographic data and clinical patient characteristics at the time of genotypic analysis.

Severity of LSCD	Patient number	Age (years)	Gender	Eye	Cause of LSCD	Preop VA	Postop last VA	Postoperative immunosuppression			Hx of rejection
								Starting Med	Ongoing Med	Duration of med (months)	
Subgroup											
Total	1	35	M	OD	Aniridia	6/152	Fc1'	MMF 2 g/day	MMF 1 g/day	12	N
Total	2	27	F	OD	Aniridia	6/192	6/48	MMF 2 g/day	MMF 1 g/day, CSA 2 mg/kg/day	13	N
Total	3	54	M	OD	SJS	Fc1/2'	6/15	MMF 2 g/day	MMF 2 g/day	30	N
Total	4	59	F	OS	Chemical burn	HM	6/96	MMF 2 g/day	N	12	N
Total	5	33	F	OD	TEN	Fc2'	6/60	MMF 2 g/day	MMF 500 mg/day, CSA 2 mg/kg/day	24	N
Total	6	36	F	OD	Idiopathic	6/240	6/15	CSA 3 mg/kg/day	CSA 3 mg/kg/day, MMF 2 g/day	42	Y
Total	7	34	M	OS	Chemical burn	HM	6/38	CSA 5 mg/kg/day	N	11	N
Total	8	71	F	OS	Idiopathic	6/60	6/24	CSA 4 mg/kg/day	N	56	N
Subgroup											
Partial	9	66	F	OD	SJS	HM	Fc1'	MMF 2 g/day	MMF 2 g/day	12	N
Partial	10	30	M	OD	Chemical burn	6/6	6/7.5	CSA 4 mg/kg/day	CSA 1 mg/kg/day	47	N
Partial	11	49	M	OD	Chemical burn	6/24	6/12	CSA 5 mg/kg/day	N	5	N
Partial	12	69	F	OS	Idiopathic	6/60	6/9.5	CSA 3 mg/kg/day	N	6	N

Preop VA, preoperative visual acuity; Postop last VA, postoperative last visual acuity; Starting med, starting medication at the perioperative time; Ongoing med, ongoing medications at the time of genotypic analysis; Duration of med, total duration of medications before the genotypic analysis; Hx of rejection, history of graft rejection before the genotypic analysis; M, male; F, female; OD, right eye; OS, left eye; SJS, Stevens-Johnson syndrome; TEN, toxic epidermal necrolysis; Fc, counting finger; HM, hand motion; CSA, cyclosporine A; MMF, mycophenolate mofetil; Y, yes; N, no.

**Figure 1:**
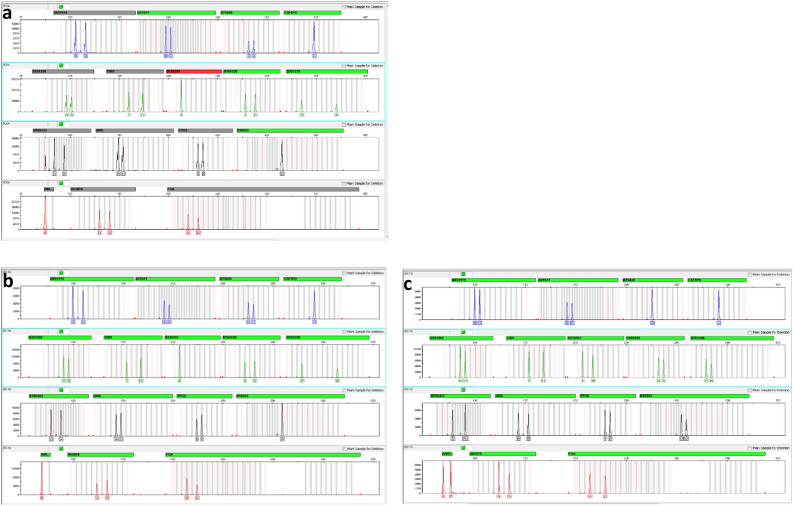
Representative results of genotypic profiles at 16 loci. The vertical axis shows the amplitude of florescence intensity of the polymerase chain reaction product and the horizontal axis shows the DNA fragment size in base pairs. The genotypic analysis of the transplanted cornea (**a**) of patient 3 at 30 months after allo-SLET is interpreted as a donor genotype when the genetic match was found with his donor reference (**b**) not his own recipient reference (**c**).

The DNA genotypic results of all patients after successful allo-LET are shown in Table 2. The genotypic results of the transplanted corneas were categorized as having a donor genotype (1 eye, 8%), mixed genotype (3 eyes, 25%), and recipient genotype (8 eyes, 67%). Representative genotypic reports from the 16 DNA loci are shown in Fig. 1.

**Table 2:** Genotypic profiles and patient results after Allo-LET.

Severity of LSCD	Patient number	Surgery	Area of allo-LET	Postop time (months)	Donor	%Donor by IC	%Donor by PK button	Genotypic interpretation	IVCM	ICIF
Subgroup										
Total	1	SLET	Total	12	Brother	79.3	55.1	Mixture	Total cornea	Total K12
Total	2	SLET	Total	13	Brother	65.9	–	Mixture	Total cornea	Predominant K12
Total	3	SLET	Total	30	Daughter	100	–	Donor	Total cornea	Total K12
Total	4	SLET	Total	21	Daughter	–	89.6	Mixture	Total cornea	Total K12
Total	5	SLET	Total	24	Sister	0	–	Recipient	Total cornea	Predominant K12
Total	6	SLET	Total	42	Sister	0	–	Recipient	Total cornea	Total K12
Total	7	CLET	Total	107	Cadaver	0	–	Recipient	Total cornea	–
Total	8	CLET	Total	138	Cadaver	0	–	Recipient	Predominant cornea	–
Subgroup										
Partial	9	SLET	Partial	12	Daughter	0	–	Recipient	Predominant cornea	Predominant K12
Partial	10	SLET	Partial	47	Sister	0	–	Recipient	Total cornea	Total K12
Partial	11	CLET	Partial	106	Cadaver	0	–	Recipient	Total cornea	–
Partial	12	CLET	Partial	112	Cadaver	0	–	Recipient	Total cornea	Predominant K12

Postop time, postoperative time at the genotypic analysis.

Among 4 eyes in the subgroup with partial LSCD and the partial limbal areas that were transplanted, all eyes were classified as having a recipient genotype. Interestingly, among the 8 eyes in the other subgroup with total LSCD with total limbal areas that were transplanted, 1 eye was classified as having a donor genotype, but 3 eyes appeared to have a mixed genotype, and 4 eyes appeared to have a recipient genotype. The eyes with the recipient genotype in the total LSCD subgroup surprisingly had successful outcomes validated by total or prominent corneal epithelia in vivo confocal microscopy or K12 by ICIF.

Among the patients with residual donor cells (donor and mixed genotypes), 3 of the 4 patients were receiving systemic immunosuppression between 12 and 30 months.

**Figure 2:**
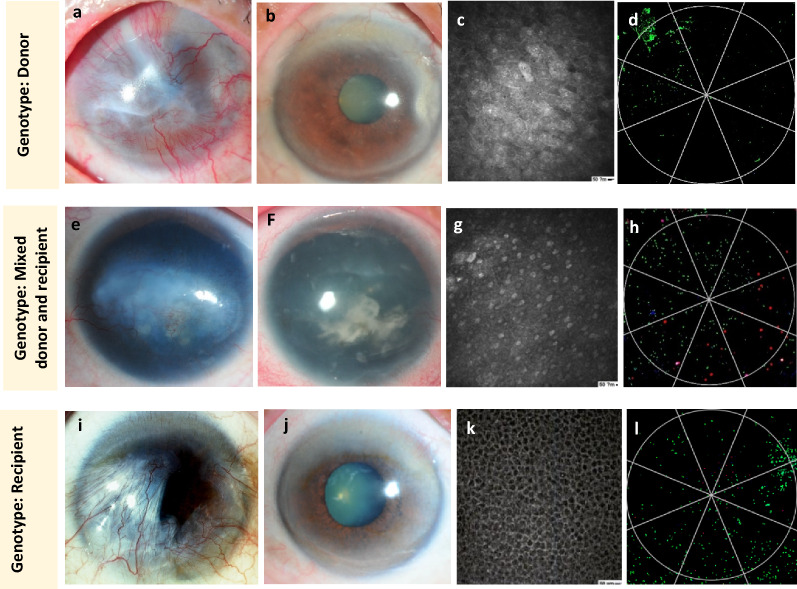
Representative clinical findings of each category of genotypic results by slit-lamp examination. IVCM shows multilayer corneal epithelium or basal corneal epithelium, and IC with immunofluorescence shows total or predominant K12 (CK12 and CK7 stained green and red, respectively) at the central corneal 5-mm zone. The results include donor (patient 3, **a**–**d**), mixed (patient 2,** e**–**h**), and recipient (patient 12, **i**–**l**) genotypes. The images in the left column show the preoperative findings of those patients with total (patient 4, patient 3) and partial (patient 11) LSCD who underwent allo-SLET (patient 4, patient 3) and allo-CLET (patient 11). The images in the other columns show the postoperative findings at the time of genotypic analysis, which show a clear central cornea with the corneal epithelial phenotype in all categories of genotypes.

**Figure 3:**
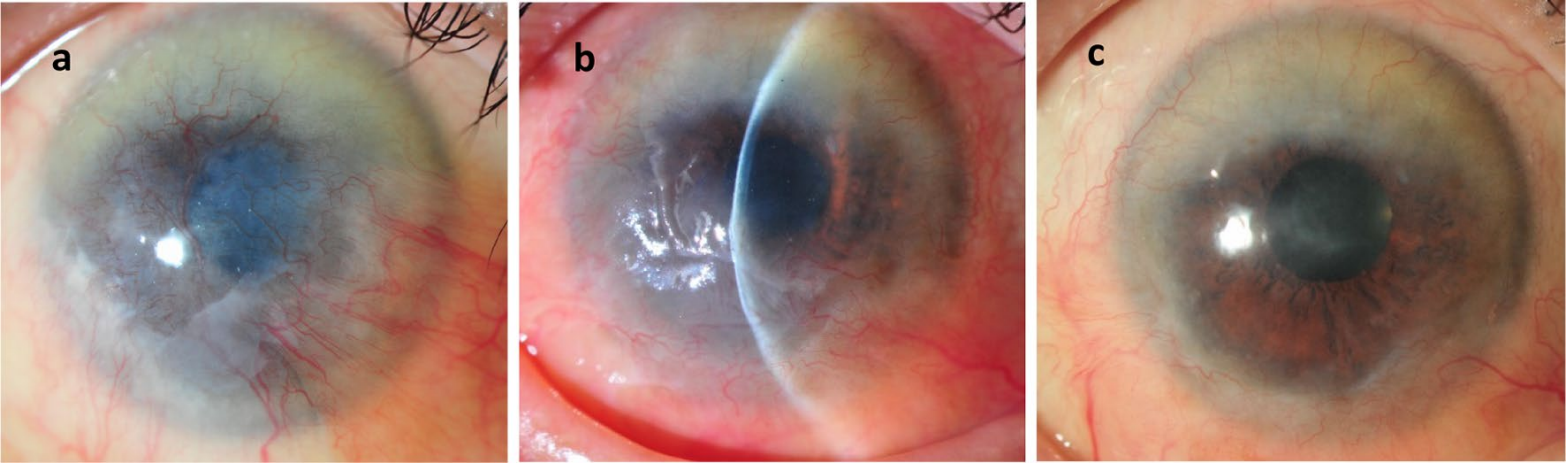
Representative images of a patient with a history of acute allograft rejection. Patient 6 had total idiopathic LSCD (**a**). After allo-SLET, she developed acute graft rejection at 32 months postoperatively with inflammation of the perilimbal vessels and corneal epithelial haze (**b**). The rejection recovered before the genotypic analysis at 42 months postoperatively with the recipient genotype (**c**).

The representative clinical findings of the ocular surface at the time of genotypic analysis are shown in Fig. 2. Despite the 3 different genotypes, the resultant clear central corneas with the corneal epithelial phenotype were identically apparent. Patient 6 had a history of graft rejection at 32 months postoperatively, at which time the immunosuppressive medications were started again and the rejection resolved successfully before recruitment into the study at 42 months postoperatively (Fig. 3).

## Discussion

After the introduction of the advanced surgical techniques of allo-LET for bilateral LSCD in recent decades, clinically successful outcomes have consistently been reported worldwide for CLET^13–18^ and SLET^3,14^. However, few studies have reported the survival of donor cells over the long term.

The current study included patients with total and partial LSCD. Of the patients with long-term survival of the donor cells, all had total LSCD and all underwent SLET. Actually, the donor genotype remained in the transplanted cornea in half of the patients with total LSCD including 38% (3 of 8 eyes) with the mixed genotype and 12% (1 of 8 eyes) with the donor genotype. Moreover, the donor cells exceeded 50% in all patients with the mixed genotype and 100% in the eyes with the donor genotype.

The extent of LSCD and surgical techniques and other factors that follow might entirely affect the results.

### SLET vs CLET

The longest time that the donor cells were detected was 30 months postoperatively after CLET or SLET. All patients with long-term survival of the donor cells underwent SLET. This result supported by the only two previous reports^11,12^ that studied the fate of the donor cells after successful allo-CLET or allo-SLET and showed that CLET failed to demonstrate long-term survival of the donor cells. Chen et al*.*^12^ did not detect any donor cells after long-term allo-CLET (mean follow-up, 22.1 months), and Daya et al*.*^11^ detected donor cells up to 7 months after allo-CLET (mean follow-up, 28 months). Only the absence or presence of the donor cells without mention of the specific amounts or percentages was reported in those studies.

Compared to the traditional surgical procedures, the studies on KLAL and CLAL tended to report longer donor cell survival times that varied from 3 to 56 months^19–22^. These results might not be applicable to the recent CLET and SLET techniques, which require only small amounts of transplanted donor tissue. Conversely, the traditional procedures used much larger sizes of donor tissue, thus providing more donor LSCs at the beginning. In addition, the amounts or percentages of the remaining donor cells were not reported in any previous studies, so we could not compare the current results.

The differences between the SLET and CLET surgical techniques may have been a factor in donor cell detection. SLET is similar to KLAL regarding direct transplantation of the stem cells onto ocular surface, which may result in outcomes similar to KLAL. The multiple steps during the cultivation process also may have interfered with the survival of the original stem cells instead of the 1 step of biopsy harvest and transplantation during SLET.

### Living-relative donor vs cadaveric donor

Using living-relative donor (ABO group matched) and performing cell expansion on the patient’s ocular surface during SLET, instead of using cadaveric donor and cell expansion in the laboratory during CLET, may result in different donor survival outcomes.

### Partial LSCD vs total LSCD

Interestingly, despite that all current patients had a successful clinical outcome, the recipient genotype was found in most patients (8 in 12 eyes, 67%). A possible explanation is that the donor grafts themselves may not be the only LSC source in the repopulation of the corneal epithelial cells but may have other roles, especially in providing a niche for the patients’ own cells to repopulate. Those effects of the stem cell may be enhanced by the properties of the amniotic membrane, which also was used in all eyes^23,24^. A previous study found that adult stem cells had tissue-protective mechanisms against immunologic reactions and apoptosis^25^. Moreover, a murine study showed that a small population of LSCs regulated the immune reaction by inhibiting lymphocyte proliferation and altering cytokine production and highly expressed genes for various anti-apoptotic proteins^26^. This explanation might apply especially to patients with partial LSCD when the donor graft helps support the patient’s remaining stem cell to repopulate the corneal epithelium, which gradually grows and eventually replaces the decaying donor cells, and maintain a normal ocular surface^27^.

The previous statements might be confirmed by the finding that all current patients with partial LSCD had a recipient genotype after either CLET or SLET. Another explanation for this result of partial LSCD might be the different surgical techniques with different amounts of donor LSCs transplanted at the beginning. In contrast to the surgery in which 360 degrees of the fibrovascular tissue on the cornea was removed and transplanted, the surgery for partial LSCD was performed only on certain areas of the diseased corneas and limbus, so fewer donor cells were transplanted.

For the patients with a recipient genotype who presented with total LSCD, the mechanism that maintains the long-term clinical successful outcome without evidence of viable stem cells remains to be elucidated, i.e., the reason for why the surface remained clear and the epithelial phenotype still demonstrated corneal epithelial cells by IVCM and CK12 marker by ICIF. Previous studies^28,29^ have reported preliminary results that autologous conjunctival epithelial cell cultivated on amniotic membrane in a suitable environment can differentiate into the corneal epithelial phenotype and maintain a healthy ocular surface after transplantation in patients with total LSCD. Alternately, the circulating donor DNA of the graft cells may play a role in chimerism similar to solid organ transplantations, which was indicated in animal studies^30,31^ and in one study of patients after kidney transplantation in whom the donor DNA was detected in recipient whole blood at 2 years postoperatively^32^.

### Sampling method of IC vs tissue from corneal buttons

The possibility to identify the donor cells may be limited partly by the specimen collection techniques. To illustrate, even though IC is minimally invasive and repeatable and used in most cases, the technique collected only the superficial cell layers. The LSCs, however, were mostly in the basal layer of the corneal epithelium^33^. An examination of all layers of corneal epithelial cells would be ideal and specimen collection from a corneal button might be more reliable. This was achievable in only two patients who underwent a subsequent PK and the donor cells were seen in both corneal buttons with a mixed genotype.

### Postoperative duration

The postoperative time may be the other factor that affects donor cell longevity. In this study, the donor cells were detected in all patients for 2 years, after which they were not detected in all cases except for 1 case after SLET in which the donor cells were detected at 30 months. The Moreover, while the donor cells were detected up to 30 months, the availability tended to fade, especially after 2 or 3 years. The patient outlier may be explained by the discussion regarding the contributory factor of partial LSCD. The finding was longer than in the previous reports of Daya et al.^11^, which was 7 months and Chen et al.^12^ in which the cells were not detected at any time. The difference might have resulted from the different techniques of SLET and CLET as discussed previously.

### Ongoing vs discontinuing systemic immunosuppression

The use of postoperative systemic immunosuppressive agents may be another explanation. While Chen et al*.*^12^ used only a short course of systemic steroid and stopped within 4 months, in the current study immunosuppressive medications (mycophenolate mofetil and/or cyclosporin) were administered for a mean of 22.5 months. Daya et al.^11^ also used long-term cyclosporine 2–3 mg/kg and the cells were maintained indefinitely.

Logically, graft rejection destroys donor cells and, thus, inhibiting graft rejection using immunosuppressive agents might prolong donor cell survival. Most current patients with remaining donor cells in this study still were still receiving systemic immunosuppression. However, among patients who had ongoing systemic immunosuppression at the time of genetic analysis, the donor cells disappeared with the recipient genotype in 4 of 7 patients (57%), suggested that the donor cell population may be depleted despite ongoing immunosuppression. Mills et al.^34^ found in an animal study that immunosuppression prolonged graft survival but not donor cell survival. They hypothesized that although clinical graft rejection was avoidable with immunosuppression, subclinical rejection was ongoing and continued to slow eradication of the donor cell population. In clinical practice, a recent suggestion was continuation of immunosuppressive agents for longer periods after a minimum of 6 months following allogenic CLET and SLET^35,36^. Considering the long-term recognition of the donor genotype at 30 months after allo-SLET in this study, with 1 patient (patient 6) with acute allograft rejection at a similar timeline of 32 months, we suggest continuing the immunosuppressive agents for at least 2–3 years postoperatively.

Finally, the accuracy in interpreting the results was a concern. A previous publication that studied a technique to detect donor cell survival after allo-LET reported a false positive result in the negative controls^37^. To minimize contamination, the protocol in the current study included using a sterile technique during IC and coupling every collection with a negative control, leaving another IC imprinting instrument in the air. This study found no false positive results in any negative controls.

A study limitation was the small number of patients. Even though the 12 eyes are sufficient to interpret the results, there are multiple confounding factors that may have affected the outcome measures. A larger number of cases with a more homogenous group of patients regarding the underlying cause and extent of LSCD and the surgical technique should be studied to analyze the survival and other related factors of donor survival such as HLA matching of donors and recipients and the specific and type/duration of immunosuppressive agents. The strengths of this study were the long-term period (up to 10 years) of investigation of the donor survival in successful allo-LET cases using recent techniques of specimen collection and genetic analysis and the ability to demonstrate the longest persistence of the donor cells of 30 months after allo-CLET and allo-SLET.

In conclusion, although the donor cells were detectable during the early years postoperatively, they disappeared in most successful cases. The essential role of the LSC donor in promoting corneal epithelial repair should be studied further to explain those clinically successful cases. The long-term use of postoperative systemic immunosuppressive agents was encouraging.

## Data Availability

The data from the current study are available from the corresponding author on reasonable request.
